# The Desirable Systemic Uncertainty in Complex IoT Sensor Networks—General Anticipatory Foresight Perspective

**DOI:** 10.3390/s22051698

**Published:** 2022-02-22

**Authors:** Andrzej Magruk

**Affiliations:** Faculty of Engineering Management, Bialystok University of Technology, 45A, Wiejska Street, 15-351 Bialystok, Poland; a.magruk@pb.edu.pl

**Keywords:** anticipation, system, future, uncertainty, knowledge, foresight, Internet of Things

## Abstract

A wide methodological spectrum regarding future research is offered by anticipation studies, with a special role of foresight studies. Many studies of this type focus on generating the desired future, taking into account the fact that it is accompanied by uncertainty. The author of this publication postulates treating uncertainty as an equivalent—in relation to the future—research object. This approach allows us to formulate general assumptions for a model of the anticipatory management of systemic uncertainty in IoT networks. The goal of such a model will not be to eliminate or even minimize uncertainty, but to regulate it to a desired level. Such an action can bring many more benefits than trying to zero out uncertainty. On the general side, uncertainty can be studied in two ways: (1) as an abstract idea, or (2) as a feature of a particular structure, also with elements of research on its abstract component. In this paper the attention is focused on the second approach. The main research area is the IoT network in its broadest sense, with a particular role of the social construct, in the context of the study of systemic uncertainty in relation to selected anticipatory actions. The overarching goal is to define a desired state, or to determine what such a desired state is, of anticipatory IoT uncertainty.

## 1. Introduction

Currently, the importance of the complex systems paradigm is a growing phenomenon. In the traditional view, a complex system is a system with multiple parts interacting, usually on a feedback basis, and affecting the subsequent new properties and behaviors of that system. Such a system has an important component in the form of unavoidable uncertainty conditioned by the following reasons: (1) the inability to measure the initial conditions of the system with infinite precision [1], (2) the difficulty of determining the ideal/expected value of such a system, and (3) the difficulty of accurately determining the future state of the system.

The main research object in this paper is the systemic uncertainty of the complex Internet of Things (IoT) sensor network as a subsystem of Industry 4.0 [2]. The high level of developmental dynamism of the IoT network determines the study of this area mainly in the future context—on the principle of feedforward correction capabilities. The unknown nature of the future introduces the study of uncertainty as a possible research topic for different technologies (in addition to ambiguity and ignorance) in the areas of foresight and anticipation [3].

According to [4], uncertainty of IoT sensor networks becomes an important aspect of their adoption, determining their: (1) suitability for the purpose of reproducing certain physical phenomena, (2) perceived quality, and (3) usefulness of the information they provide.

According to J. Derbyshire, the state of uncertainty of the future forces the use of anticipatory actions based on analyses of alternative options, also based on ignorance. Creating the future only on the basis of methods using past data is highly dangerous. It includes important but secondary aspects of the future [5].

The study mainly focuses on the area of systemic uncertainty, and not environmental uncertainty, because that systemic uncertainty is a controllable uncertainty from a social point of view. The overarching goal of this paper is to establish the desired state of anticipatory systemic uncertainty of IoT, the existence of which is determined by [4]: (1) epistemic uncertainty, (2) ethical uncertainty, and (3) utilitarian uncertainty.

Epistemic uncertainty (also called endogenous uncertainty) is based on the internal knowledge (or ignorance, depending on the research perspective) of the system caused, for example, by limitations of the measurement equipment and in the social consensus—dependent on the observer/stakeholder of such a system. This uncertainty may be related to the knowledge of the state not only in the future, but also in the past and present. This uncertainty is susceptible to discovery and expansion of knowledge, and can be directly influenced by an individual [3,4,6,7]. Ethical uncertainty arises from the observer’s own attitude/trust toward other entities, treated as systems, people, or technical devices. Utilitarian uncertainty refers to a forward-looking perspective. It is based on the belief that there is knowledge that either does not exist or whose existence is not even anticipated [4].

According to [4], the most significant challenge, when managing IoT networks—in which uncertainty is an integral component—is to change uncertainty in accordance with the changes in social needs on which the functioning of IoT networks is based. A key challenge in this area is therefore the organization of the process of such changes. According to the author of this publication, the organization of the process of changes in social needs can take place through anticipatory foresight systems, based on selected research methods. Anticipatory foresight systems should be treated as the main representatives of future research based on social construct taking into account: (1) the aspect of uncertainty with the possibility of its modification, (2) the available, unavailable, and unobtainable knowledge, as well as the attitude to it, and (3) the futures in their different types. The author of this article proposes the use of a model in which a change in uncertainty can take place through its understanding and modification to a desired level.

The rationale behind the validity of the above approach is the position of R. Poli, according to whom uncertainty is the prediction of anticipatory systems. It helps to develop more sophisticated courses of action and is required to understand many individual and social behaviors. Also, it is characteristic of the behavior of relevant complex systems. All sources of uncertainty related to the evolving present and past must also be related to futures studies. According to S. L. Brown and K. M. Eisenhardt, looking into the future is a complex and conflictual process of analyzing, experiencing, interpreting, and absorbing uncertainty. Futures studies, represented herein through anticipation and foresight research, is a field that lies between basic ignorance of the future and an effort to use the future to make decisions and strategies in the present [3].

To help understand the concepts of uncertainty, knowledge, and the future, this study uses formulations, definitions, and tools specific to conceptual modeling.

## 2. The Relationships among the Concepts of Uncertainties, Anticipation, and Foresight Studies

A proper understanding of anticipation requires the adoption of an innovative conceptual framework [8]. Anticipation, according to R. Poli, is a response to complexity. Complex systems are inherently uncertain. This uncertainty arises in part because the features of the system interact in ways that allow small, undetectable, or difficult-to-detect changes to have dramatic effects [9]. According to M. Nadin, anticipation is “driven by information processes that occur in a context of incomplete information and uncertainty”. T. Fuller, on the other hand, suggests that anticipation is a mediating process between knowledge and action [10].

Anticipation is the coupling between a system and its environment and anticipation is a cognitive projection [3]. In relation to the IoT system and its systemic uncertainty, the definition of anticipation should be understood as the coupling between the IoT sensor network and its environment and as an epistemic component.

The above definitions of anticipation refer to the so-called weak type of anticipation, i.e., one that is based on a cognitive model developed by the anticipation system itself [11].

According to M. Nadin, an anticipatory system is a system with a present state that not only depends on past experiences, but also on possible future realizations [10]. The best-known definition of anticipatory systems is the one proposed by R. Rosen: “An anticipatory system is a system containing a predictive model of itself and/or its environment, which allows it to change state at an instant in accord with the model’s predictions pertaining to a later instant” [11]. Relative to the topic of this paper, an anticipatory IoT sensor system is a system that makes decisions in the present according to predictions. According to the author, these predictions and decisions may result from developed desired uncertainty based on a desired future. The desired future—concerning something that should, or that might eventually happen—must be the result of a social construct based on a particular kind of knowledge and/or ignorance. The desirable future should therefore not be dominated by quantitative approaches [12], although it should incorporate these studies.

In the author’s opinion, the methodology that takes into account the above assumptions is the qualitative-quantitative methodology of foresight research with its wide, and at the same time open and flexible, research apparatus. This attitude also corresponds to the concept of systemic anticipation. According to its assumptions, the best possible anticipation is achieved through the knowledge of what elements the studied phenomenon consists of and what relations occur there, creating a new studied whole [12].

The foresight’s view of predictive uncertainty gives the following attributes to scientific knowledge: absent, incomplete, irrelevant, insufficient, inaccurate, ambiguous, equivocal, inconsistent, fragmented, manipulated, complex, or otherwise limited [13]. Regardless of the condition or attribute, knowledge by its nature is always a social construct [6].

Anticipation in futures studies is also called “Foresight 2.0”, or “design-based foresight”. It builds on the results derived from forecasting and foresight models, and aims to implement them into decisions and actions [1].

Foresight is a deliberate, critical, reflective, and creative engagement in future action-dependent states of affairs [3]. According to R. A. Slaughter, “foresight is a human attribute that allows us to weigh up pros and cons and to evaluate different courses of action. Moreover it help us to invest possible futures on every level with enough reality and meaning to use them as decision making aids. The simplest possible definition of foresight is: opening to the future with every means at our disposal, developing views of future options, and then choosing between them” [14].

Anticipation shares some of the characteristics of foresight, namely unpredictability, a qualitative nature, and a focus on discontinuities. The distinguishing features of anticipation in futures studies are those that are becoming known as “futures literacy”, along with the full acceptance of complexity. The two main elements of futures literacy are the classification of future types and the classification of uses of futures. In the case of this paper, this is done by incorporating futures typology analysis, uncertainty scopes analysis, and levels of knowledge [1].

Referring more specifically to the research [10], the relationship between foresight and anticipation can be described as follows: (1) foresight as a structured social activity [14,15] based on specific methods concerns what you do in order to foresee the future, while anticipation is about how you apply the methods of foresight, (2) the foresight process includes anticipation, (3) anticipation is part of foresight and can result in anticipatory action, and (4) anticipation can be triggered by some foresight methods.

M. Kienegger, M. Hörlesberger, and S. Giesecke further suggest that the depth of anticipation is related to “the effectiveness of the methods used in changing the assumptions of stakeholders of the future”. J.P. Bootz considers anticipation in relation to foresight research as a basic cognitive activity. R. Poli argues that anticipation has two key elements: foresight and enactment on foresight [10].

According to R. Poli, we also have to deal with the following distinction between foresight and anticipation: foresight affects the generation of cognitive strategies that system A develops in order to understand the future of some other system B, of which A may or may not be a component. Anticipation, on the other hand, is a property of the system, intrinsic to its functioning [11]. In the case of this paper, the IoT sensor network acting as a component of a social construct belongs to system A. Industry 4.0 as the environment in which the IoT sensor network operates belongs to system B. The environment of Industry 4.0 is a broadly defined network of socio-economic-technological dependencies.

The future is often constructed as a linear continuation of the past and present, partially stripping it of its complex and unexpected character. A commitment to linearity narrows the range of plausible imagined futures and provides a false sense of certainty. The foresight approach avoids trying to know the ontologically unknown future. Instead, it attempts to generate a preferred future based on narratives, expectations, memories, and imaginings, among other things [16]. Using new futures to discover new ways of making sense of the emerging present is one way of using the unknowable as it begins to become knowable, increasing the ability to discover the present [1].

There is a wide range of knowledge, both explicit and implicit in different time frames, influencing the formation of different types of futures and perceptions about them. The analysis of different futures requires new experiences, new ways of combining known elements, and different tools supporting this type of reflection. In foresight terms, discovering the future involves seeing what is known as new, or even strange, by engaging from different perspectives. Efforts to control, manage, and design the future create unprecedented uncertainty that should also be thoughtfully controlled, managed, and designed [16].

## 3. Systemic Uncertainty vs. Internet of Things

**Figure 1:**
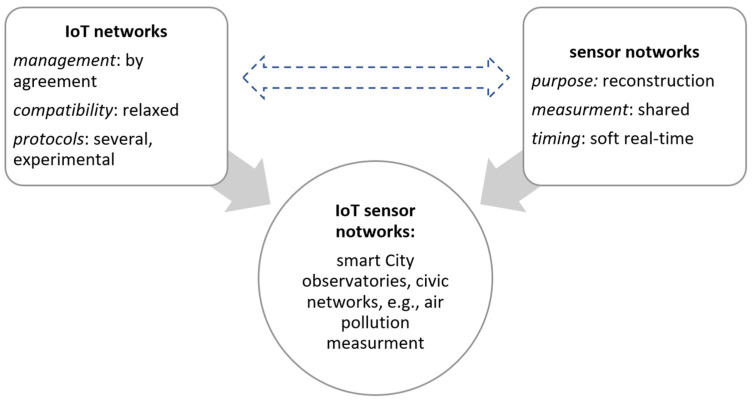
The main important elements of IoT sensor networks. Source: [4].

An IoT sensor network is a specific combination of technology, management, and application domains (Figure 1), specifically designed to satisfy the desires of the observer (and the social system, represented by the observer) regarding both the phenomenon and the way it is to be measured. 

W. Bojarski in 1981 made an in-depth analysis of uncertainty regarding complex systems. Despite the huge technological gap between the systems of 40 years ago (which were not even based on Internet networks) and the current complex IoT systems, the validity and importance of the following aspects of system uncertainty should be emphasized [17]: (a) the great number of elements in the system, their high heterogeneity and variability over time, (b) the very large number of possible states, functions of the system and its individual elements, (c) the difficulty of recognizing the states and relationships of the subsystems interdependent with the analyzed system, (d) the impossibility of effective management of the complete system resulting from limited possibilities of evaluating the significance of various states of the system and possible errors in the retrieval, transmission, processing, and aggregation of information generated in the system, (e) the lack of practical possibility of full representation of the system in the form of a model, and (f) the impossibility of determining the full future state of the system resulting from, among other things, the huge number of possible combinations of information generated by the system components.

In relation to the research of A. Furlanetto and R. Poli [5], in the case of IoT networks the problem is not the lack of information. The problem is the uncertainty of uniform structure, compatibility of information from different sources (sensors come from different manufacturers), and the danger of false information. In addition, in the case of large complex networks, such as the IoT, it is necessary to remember the potential uncertainty resulting from the excess of information, not its deficiency.

In the context of IoT sensor networks, uncertainty arises in the measurement of epistemological situations, where the probability distribution of the discrepancy between the true (expected) value and the measured value is not known, or where utility is not assignable. Some aspects of such uncertainty may be attributable to the structure of the instrument, while others may be attributable to the observer and the observer system, where the sources of uncertainty may be multiple. In both cases, uncertainty may arise from [4,18,19]:

- Different perceptions, understanding, and modification of uncertainty by different participants in such a system,
- Different origins and quality of sensors, measurements, and instruments,
- Flaws in IoT implementation caused by lack of device compatibility,
- Violation of the privacy of users,
- Security issues—e.g., IoT sensors are connected in event/phenomenon management systems, aiming to show the status of the system. However, it is often very difficult to interpret and distinguish which phenomena are legitimate and which are based on malicious events.

## 4. Materials and Methods

The author’s previous research was focused on the analysis of the common ground between systemic uncertainty, future, and knowledge [20,21], referring to, among others, the idea of cones of “future, uncertainty, plausibility, possibilities and everything” [22,23,24,25,26,27]. The idea of future cone has been promoted most strongly by J. Voros [15,28,29,30,31,32], whose prototype can be found in the works of Amara [33], and Hancock and Bezold [34].

The main inspiration for writing this article was the publication by P. Cofta, K. Karatzas, and C. Orlowski [4], which presents a general overview of uncertainty research in the IoT area. This publication inspired the author to conduct an in-depth analysis with three additional dimensions—relative to those listed above—in the form of: (1) IoT sensor networks, (2) anticipation, and (3) social construct.

This allowed for the creation of an innovative model of anticipatory management of systemic uncertainty.

Additional inspirations for creating the above-mentioned model and simulating its operation in relation to the IoT system treated as a social construct were the following publications [1,35,36,37,38,39].

The innovative model is based on four dimensional spaces, viz:

- Four types of future (based on the classical typology of future according to N. Henchey [28], R. Amara [28,33], T. Hancock and C. Bezold [34]),
- Four levels of knowledge (based on knowledge cycles [40]),
- Four scopes of uncertainty (referring to types of future according to H. Courtney [23,24,37]),
- Four stages of anticipation (based on four possible attitudes to the future according to R. Ackoff [38,41]).

Other typologies of future [15,28], knowledge [19,42], and uncertainty [43,44] are also available in the literature. The author is aware of the possibility of other combinations of uncertainty, future, and knowledge, covering the problem in more detail. However, the author assumes that from the point of view of usability in relation to IoT networks, the general model-based on four-point scales is satisfactory. This approach is due to the fact that the model proposed in the article is a starting one, which aims to give a general direction of thought.

P. Cofta, K. Karatzas, and C. Orlowski point out a strong dependence of system uncertainty on socially developed constructs that influence the observer’s process of observing the system [4]. According to the author, socially evolved constructs should not only be based on generally accepted “inviolable” norms, but also should themselves dynamically create the desired states of the IoT system, thus influencing the desired states of uncertainty and future of the observed system. With this approach, it is possible to create a network in which observers will be active participants in the whole system and not just passive recipients of the analyzed signals.

Regarding the idea of the desired state of systemic uncertainty, it is important to emphasize its direct relationship to the concept of the desired future characteristic of foresight research [45,46,47,48,49,50,51]. It is also one aspect of anticipation itself. The desirable future state (called by J. Voros preferable future) is a kind of combination of states of other available future types [28]. In the author’s opinion, it may be closely related also to the available or desired level of knowledge and to the uncertainty that affects the future under study. The state of desired knowledge may be knowledge that already exists and that satisfies the researcher for the moment, or it may be a state that should be achieved.

On the one hand, the paper is part of a practical postulate [4] regarding the need to develop new theories and practices regarding views of uncertainty in IoT sensor networks. On the other hand, the author was guided by the utilitarian aspect—referring to the potential consequences and negative effects of not considering the validity of uncertainty studies [35].

The kinds of decisions people make vary depending on how much knowledge or information they have about the system and the environment in which it operates. There are three conditions of decision environments: (1) uncertainty, (2) risk, and (3) certainty, depending on the probability of evaluating each possible outcome [3]. The model proposed in this paper is dedicated to complex situations/problems in the first decision environment. Thus, it can be applied, for example, in preventive systems regarding the prevention of environmental emergencies.

As uncertainty increases, we are forced to anticipate a future that is less and less real [1]. In turn, the increasing sense of a real future generates inactivity and indifference, consequently generating zero uncertainty or certainty. It should be remembered, however, that eliminating uncertainty altogether would render anticipatory activities pointless [52]. The logical consequence of this approach is to maintain, regulate, and even create [7] uncertainty at the appropriate optimal level.

As in the publication [3], this paper focuses more on trying to understand and use the concept of uncertainty rather than on reducing it. At the same time, it is an attempt to modify uncertainty in anticipatory terms resulting from not knowing the future.

According to R. Rosen, anticipatory behavior is behavior that “uses” the future in its actual decision-making process [53]. Referring to the research by R. Ackoff, the very strong or strong connection with anticipation has the so-called interactive or preactive behavior. Reactive and passive behaviors are secondary components of behavior [38], but also have an anticipatory potential, but of lesser strength.

The above-mentioned actions, passive, reactive, preactive, and interactive, are four possible attitudes to the future, according to R. Ackoff [38,41]:

- In the passive attitude (inactive) the intention is to adapt temporarily by preventing change and maintaining the status quo,
- In the reactive approach, the intention is to avoid what is not desirable (but at the same time the desired future state is not sought); the only goal is to restore the state that existed before the problem occurred,
- In the preactive approach, the intention is to minimize risks and threats, and to exploit opportunities by anticipating the future, setting goals for it and accelerating change by working toward it,
- In the interactive approach, the intention is to “create as many alternatives as possible” through various actions in the present; in this approach, the future is open-ended (rather than linear) and is created based on the identified desired present.

This paper applies an analysis of the impact on uncertainty of (1) knowledge, as the opposite perspective of ignorance, and (2) anticipation foresight activities in the form of types of future and selected research methods.

## 5. Results

**Figure 2:**
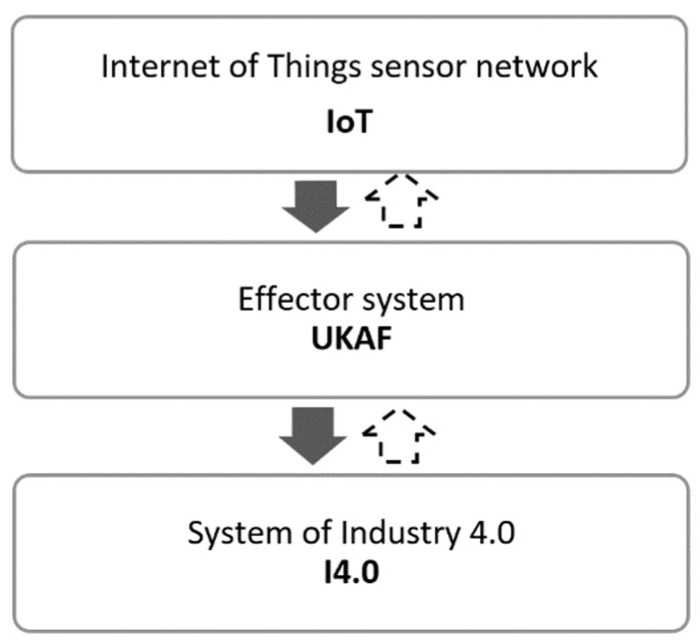
A simplified model of anticipatory management of systemic uncertainty in an IoT network. Source: [11].

Referring to the definitions of anticipatory systems presented in the previous chapters and to the research of R. Poli (based on the research of R. Rosen) presented in [11], this chapter characterizes the principle of the model of anticipatory management of systemic uncertainty in an IoT network as a subsystem of uncertainty of Industry 4.0. Figure 2 shows a schematic presentation of the simplified model.

**Figure 3:**
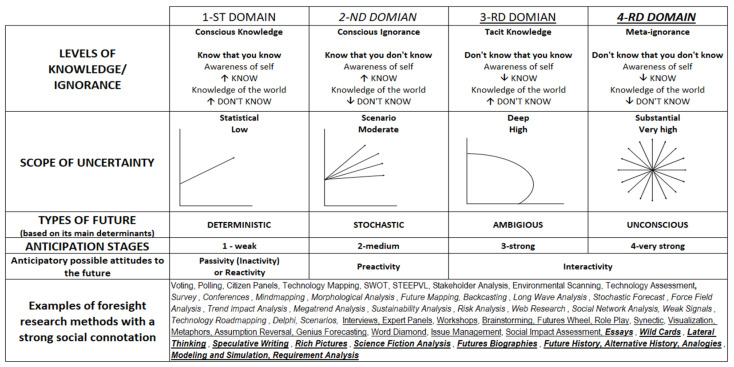
UKAF Matrix—effector system.

In the analyzed model, I4.0 is a complex dynamic uncertainty system of Industry 4.0. The second uncertainty system, the IoT, is a sensor network functioning as a measurement instrument. An important assumption is that the dynamic evolution of the IoT is faster than the dynamic evolution of I4.0. By observing the uncertainty level of IoT, we obtain information about the subsequent uncertainty state of the I4.0 system. We assume that the uncertainty of the IoT can affect the uncertainty of I4.0, and the uncertainty of I4.0 can affect the uncertainty of the IoT. The direction from I4.0 to the IoT can be seen as an update or modification of IoT uncertainty. For the IoT to influence I4.0, the IoT must be equipped with a set of effectors—in this case in the form of UKAF—a matrix of uncertainty, knowledge, anticipation, and foresight (Figure 3). This set of effectors allows the IoT to act on I4.0 in such a way as to change the uncertainty dynamics of I4.0. Consider partitioning the state space of I4.0 (and hence the IoT) into desirable and undesirable states. As long as IoT uncertainty dynamics remain in the desirable space; i.e., we are able to establish, understand, and maintain desirable states of uncertainty and the future in the IoT based on available knowledge, the IoT takes no action via the UKAF effectors. When the IoT dynamics move into an undesirable area of uncertainty (e.g., by realizing through the UKAF results that we are moving only into the area of conscious knowledge, i.e., the dangerous area of determinism), the effectors are activated to make the IoT uncertainty dynamics return to the desired state. In this case, the activation of effectors will occur through the UKAF system by using an appropriate set of foresight methods focused on the social construct (the observer/user of the system), with the goal of, for example, identifying tacit knowledge.

In relation to the model of R. Poli, the author proposes the following decomposition of all stages of model building:

1. Selecting the model of uncertainty in the IoT,
2. Selecting the control variables of uncertainty in I4.0,
3. Designing the effector system UKAF,
4. Programming the effector system UKAF,
5. Distinguishing desirable from undesirable regions,
6. Including a device to reset the model.

First stage: the model of uncertainty in the IoT is the model of systemic uncertainty of the IoT.

Second stage: the control variables of uncertainty in I4.0 have strict relationship with three components of systemic uncertainty, namely epistemic, ethical, and utilitarian, in the following areas:

- Control variables of epistemic uncertainty relate to the area of knowledge,
- Control variables of ethical uncertainty are characteristic of the social construct,
- Control variables of utilitarian uncertainty are the most numerous and refer to futures studies, foresight methodology, and anticipation.

Stages three, four, and five have a strict connection with the UKAF effector system (Figure 3) and are described in following paragraphs.

The main purpose of the UKAF effector system is to activate a specific foresight methodology in the event of an undesirable state of uncertainty in the IoT system based on, among other things, knowledge built on the results of IoT sensor readings and the undesirable future and anticipation stage that the IoT network relies on at any given time. Using appropriate methods, the IoT system (or a human through the IoT system) will be able to make optimal (desirable) anticipatory (anticipation) decisions. Desired states are determined under identified measurement uncertainty, depending on the possessed knowledge (and the desired future state of the I4.0 system), which is closely related to future types and the social construct.

An additional goal of UKAF is also to activate interactive and/or reactive actions in the IoT system (treated as a social construct), while attempting to supplement or minimize reactive and passive actions that are more characteristic of traditional risk-based models [5].

The first domain is characteristic of small networks and concentrates on the high level of knowledge possessed by the IoT system and awareness of it (known knowns, conscious knowledge). There is a high level of awareness of self and knowledge of the world (know that you know). The values from the full sensor readings are known and the expected values are known, and the decisions to be made based on those results are known, both now and in the future. At this level we are dealing with low uncertainty, otherwise known as statistical uncertainty or known certainty. This domain is characterized by a deterministic and clear-enough future. This future has characteristics similar to probable future (perceived as an extension of the present) and plausible future (which we can imagine, given currently available knowledge), according to R. Amara, and/or projected (extrapolated) or predicted (that someone claims “will” happen) future, according to J. Voros. This future can be determined on the basis of not very complex research, e.g., forecasting. Characteristic foresight methods with strong social connotation for this group are voting, polling, citizen panels, technology mapping, SWOT, STEEPVL, stakeholder analysis, environmental scanning, and technology assessment. In this case, the social construct tends to adopt an attitude of passivity and inactivity (reactivity only when there is a problem), with high confidence in the effectiveness of quantitative research methods. Time is treated in a linear way as a limited resource. System operation is based on known patterns—based on past data which is far from anticipatory behavior. On the four-grade scale it takes the value of 1—which means a weak degree of anticipation. Only when a problem is encountered is its cause or source identified and an attempt is made to remove or mitigate it. If this approach is successful, the system returns to the state before the problem occurred.

The second domain is based on the IoT system’s high level of awareness of its ignorance (known unknowns, conscious ignorance). There is a high level of awareness of self and high ignorance of the world (know that you don’t know). There is a relatively high level of confidence in the results being read while knowing that the true expected value is unknown, but that a range of all alternatives is possible. At this level we are dealing with low but moderate uncertainty, otherwise known as scenario or known uncertainty. This domain is characterized by stochastic futures. This future is a kind of combination of the characteristics of the possible future, according to R. Amara and J. Voros. The stochastic future is dependent on future knowledge that is not currently available but can be developed over time. The future can be described as one of a few discrete scenarios without the knowledge that will occur, but this future is always better than the present. Characteristic foresight methods with a strong social connotation for this group are survey, conferences, mind mapping, morphological analysis, future mapping, backcasting, long-wave analysis, stochastic forecast, force field analysis, trend impact analysis, megatrend analysis, sustainability analysis, risk analysis, web research, social network analysis, weak signals, technology roadmapping, Delphi, and scenarios. In this case, the social construct takes a preactive stance. The operation of the system is based on anticipating alternative futures, setting goals for them, and acting toward them. Time is treated as an opportunity cost. On a four-point scale of anticipatory behavior, it takes a value of 2—that is, a medium degree of anticipation. Several alternatives identify a potential problem by identifying its impact and proposing ways to solve it.

The third domain concerts on the low level of possessed awareness of the knowledge possessed by the IoT system (unknown knows, tacit knowledge). There is a low level of awareness of self, but a high level of knowledge of the world (don’t know that you know). The range of expected states (outcomes) of several IoT system models is known, but there is no awareness that the values from the readings may be incomplete or untrue. This can lead to a misconception that all interpretations of the readings are correct. What follows is a difficulty in understanding reality, an inability to perform critical analysis on the basis of existing knowledge, resulting in potential misuse of that knowledge. At this level we are dealing with high uncertainty, otherwise known as deep uncertainty or uncertainty of known information. Ambiguous future is characteristic in this domain. This future partly overlaps with the characteristics of the possible future according to Amar and the preposterous future according to J. Voros, which is seen as a future that is very difficult (even absurd from today’s point of view), but ultimately possible, to imagine. The range of these futures can be determined by a limited number of key variables. Time is treated in a fuzzy manner. Typical foresight methods with a strong social connotation for this group are interviews, expert panels, workshops, brainstorming, futures wheel, role play, synectics, visualization, metaphors, assumption reversal, genius forecasting, word diamond, issue management, and social impact assessment. In this case, the social construct adopts an attitude of illusory certainty about outcomes and satisfaction with the way things are. On a four-point scale, anticipatory behavior takes on a value of 3—that is, a strong degree of anticipation.

The fourth domain concerts on the zero level of knowledge possessed by the system (as a social construct) IoT and awareness of it (unknown unknowns, meta ignorance). There is zero-level awareness of self and knowledge of the world (don’t know that you don’t know). The full-reading values are unknown because of a lack of awareness of the existence of new data sources, e.g., from “private” sensors, and the expected values are unknown. This is the state of not knowing at a given point in time, but it is not completely unknowable. At this level we are dealing with multiple dimensions of very high uncertainty, otherwise known as substantial uncertainty or unknown uncertainty. This domain is characterized by an unconscious (true ambiguous) future. This future partially overlaps with the characterization of potential future according to J. Voros, and includes everything beyond the present moment. Because we are dealing with a nonpredictable range of outcomes, this future should be created on the basis of complex, normative research that identifies and creates the desired present. In this case, there should be an understanding that the future can be created by the way we act in the present. Characteristic foresight methods with a strong social connotation for this group are essays, wild cards, lateral thinking, speculative writing, rich pictures, science fiction analysis, futures biographies, future history, alternative history, analogies, modeling and simulation, and requirement analysis. In this case, the social construct must take a strong engaged and interactive stance. The operation of the system is based on unknown patterns, which has a strong connotation of anticipatory behavior. On a four-point scale, it takes a value of 4—that is, a very strong degree of anticipation. Time is treated in a qualitative way. In such a system, it is permissible and even desirable to question existing assumptions, making transformative decision-making changes in the present possible. Amara describes the type of future described in the paragraph above as a preferable future that we would like to live in. According to J. Voros, this type of future is based on a way of thinking: “should” or “ought to” happen. Preferable future, however, cannot be equated with unconscious future. Preferable future should result either from the existence/identification of unconscious future or from the combination of deterministic and/or stochastic and/or ambiguous and/or unconscious future.

An important assumption is that recommended foresight research methods in a given domain can also be used in other domains, but their linking power will be weaker, which may result in less-relevant results.

Let us assume three examples (two purely hypothetical and one based on real environment of IoT) of how the UKAF system works. The first case is focused on a state of low uncertainty based on deterministic approach toward the future and passive or reactive anticipatory action. At some point, the IoT system, through social activity, may become aware that the previous scope of uncertainty is an undesirable state, being burdened with, for example, too high a level of unreflective trust, which may eventually lead to unsafe situations in the whole I4.0 system. This triggers the UKAF effector to establish a different scope of uncertainty of the IoT system. The different scope of uncertainty can be a specific state, e.g., moderate or a mixture of different scope of uncertainty. If, for example, moderate uncertainty is considered a desirable state, it will force the application of several foresight methods specific to the second domain. The results of these methods should allow for identifying knowledge about what the IoT does not know, while triggering reactive anticipation of the IoT system. 

The second case is based on a very high level of uncertainty in some areas, which may result, for example, from the fact that the IoT network is managed by people with a very high level of incompetence (e.g., making decisions that are not their own, deeply trusting solutions proposed by others). The realization by the IoT system (through a social construct) of the existence of the “I don’t know that I don’t know” state as an undesirable state, will force the activation of the UKAF effector in order to determine, for example, another scope of uncertainty, which will force the application of several foresight methods characteristic for the first, second, and/or third domains. The results of these methods should allow for the identification of knowledge at four levels, triggering all types of anticipation of the IoT system at the same time. The state of meta-ignorance can also be a desired state according to H. Khahn’s principle, according to which “one should also think about the unthinkable” [15].

The third example is focused on the idea of a quantum Internet of Things (QIoT) and its security. According to S. Ghose, it is possible to create a quantum Internet, based on the teleportation of information [54]. One of the important research areas in this context is the idea of the QIoT and the security of such a network based on the concept of quantum cryptography. The convergence of the Internet, the IoT, and quantum computing has been discussed by various researchers [55,56,57,58,59], but so far no idea comes close to practical applications. One of the reasons is that we have many years to implement quantum computing on a commercial scale [60,61]. Additionally, many security issues (regarding communication, sensors, analytics, and applications) related to the QIoT network problems (concerning, for example, data breaches, irregular updates, quantum key resource allocation [55], etc.) have not yet been resolved despite the ongoing work on such possibilities. Moreover, as quantum computing is constantly evolving, there is no guarantee that developed post-quantum IoT cryptosystems (IoT systems protected from the currently known quantum computing attacks) will be resistant to novel attacks [62].

Referring to the above-mentioned topic, which is the QIoT and its security, it should be stated that we are operating in the second domain of the UKAF system, i.e., the domain in which the desired state of systemic uncertainty should be moderate uncertainty, which should be analyzed in several alternatives. This is due to the following levels of knowledge, future, and anticipation. (1) Regarding the level of knowledge, despite many studies to date, we are dealing with a high level of awareness of our ignorance. There is a relatively high level of trust in the research results so far, resulting, for example, from the fact that a given topic is discussed in the pages of many reputable journals [58,59,63]. At the same time, there is a high awareness that the true, final (expected) results are not yet known. However, it is possible to determine the range of all alternatives in a given research context. (2) The future is stochastic because it is usually analyzed in the form of several scenarios. For example, with regard to IoT quantum security cryptosystems we deal with the following alternative scenarios: pre-quantum, post-quantum, and hybrid models [64,65,66,67]. In the general area of IoT security, one can distinguish scenarios focused around cloud and blockchain, postquantum cryptography, evolutionary techniques [68], etc. Full knowledge of each of the scenarios is not available today, but by observing the research dynamics in these areas it will surely be developed over time. (3) Regarding the anticipatory attitude, we are dealing with a preactive approach where the intention is to minimize risk and threats and to take advantage of opportunities by predicting the future and setting goals by working in this direction [69,70,71,72,73]. Previous foresight studies, partially focusing on the studied area, were based on such methods as horizon scanning, an online survey among subject-matter experts [74], expert panels [75], trend impact analysis [76], and conferences [77]. Other methods that should be recommended for further analysis are future mapping, megatrend analysis, web research, social network analysis, weak signals, technology roadmapping, Delphi, and scenarios. Managers of IoT networks must pay close attention to advances in quantum computing [62]. Systematic application of appropriate foresight methods will in a while allow a revision of the state of knowledge of the topic under study and determine whether we still need to move to the area of the second domain when analyzing the uncertainty, or whether it will be necessary to transpose it to another domain. The main shortcoming of this approach is the time required to get the right results.

The property of anticipatory systems to elude the possibility of repetition by memory [78] and the developmental dynamism of the IoT (based on various social constructs) result in the fact that the desired state of such systems is not a state in the sense of a certain final numerical or qualitative value. Desired uncertainty is a kind of optimal uncertainty, whose scope is best under the given conditions of anticipatory IoT network management. These conditions are determined by (1) the found or desired level of knowledge, i.e., the epistemic state that the IoT deals with at a given moment, (2) the desired type of future (or their combination) that is or can be considered, and (3) the scope of uncertainty affecting the level of anticipation. The UKAF model abandoned the analysis of complete certainty (i.e., zero uncertainty) and the state of total irreducible uncertainty and ignorance. Due to the developmental dynamism of IoT, the first case is an impossible state. It is even a state whose assumption of desirable existence is dangerous because it risks an attitude of “sleeping” on possible threats or an attitude of “stifling” the kind of social spontaneity that leads to progress [9]. This approach severely limits the perception of perceived reality [79]. The second case should be more the domain of purely philosophical and speculative considerations, not necessarily having a real impact on network performance.

To be effective, UKAF should not be based on a single domain but on a combination of proactive, interactive, reactive, and even passive actions. Each approach implies a different way of designing a foresight methodology. Moreover, it is well acknowledged in futures research that “no single method should be trusted; hence, cross referencing methods improves foresight” [80]. Anticipation can occur in all domains in different forms. The boundaries between the four domains are fuzzy and fluid; they change over time and allow for movement from one domain to another.

Improving the performance of the anticipatory system in IoT networks involves both technical and human challenges because of the dependence of the anticipatory system on the ability of individuals and their cooperation regarding identifying, interpreting, and combining data into knowledge, and becoming aware of areas with high levels of ignorance. On this basis, it is possible to predict and, in the most ideal case, anticipate future events and their consequences.

## 6. Discussion and Conclusions

A direct author’s reference to this article can be found in [20,21]. For several years, the author has been dealing with the subject of uncertainty management in the future in the context of the development of modern technologies, in particular Industry 4.0 and the Internet of Things. In each of the publications, he presents the results of research that focuses on one or several specific aspects characteristic of the above-mentioned subject matter. The latest research is focused on building a model of uncertainty management in terms of the foresight methodology. The publication [20] outlines a general framework of the model, which is analyzed on seven dimensions, focusing on four areas: uncertainty, knowledge, future, and foresight. In publication [21], this analysis was enriched with the research on emerging technology and the special type of knowledge, which is foreknowledge. In this publication, the author’s attention is focused on forward-looking research, which allows for, for example, an analysis in the space of four possible attitudes to the future according to R. Ackoff. This approach allowed for, but also forced the construction of an innovative model based on four dimensional spaces, viz: four types of future-based on the classical typology of future according to N. Henchey, R. Amara, T. Hancock and C. Bezold, four levels of knowledge (based on knowledge cycles), and four scopes of uncertainty (referring to types of future according to H. Courtney).

Table 1 summarizes the various main ideas discussed in this paper regarding solutions to the problems presented so far, as well as possible future frameworks, both from the literature and the author’s perspective. Other considerations are presented in the paragraphs.

Based on the view of G. Berger [1], it can be concluded that the most relevant question to ask ourselves regarding actions in the face of uncertainty is the following: “How can we best prepare for an ever-changing and uncertain world and how can we choose courses of uncertain action that can achieve our preferred/desired goals?” Placing the focus on preparedness for future uncertain challenges can be facilitated by skillful anticipatory management of systemic uncertainty.

Due to the increasing complexity of IoT systems [81,82,83,84,85,86], it is difficult to unambiguously accept, e.g., in terms of security, all results, and thus to make sense of all obtained forms of information, resulting in a high degree of systemic uncertainty.

According to the author of this publication, in the face of high systemic uncertainty, managers of IoT systems are required to anticipate the actions of entities that use them (IoT). Anticipating a potential threat, protective measures can be implemented.

Paradoxically, the more difficult it is to predict the future, which is a result of, among other things, ever-increasing complexity and uncertainty, the greater the demand for predictions and anticipatory actions [87].

Since, in IoT sensor networks, the state of uncertainty causes the state of discrepancy between the true (expected) value and the measured value to be unknown, one solution may be the concept of establishing a desired value that can be modified according to societal needs and the state of knowledge. A desired value is possible through anticipatory uncertainty modeling based on foresight methodology.

Anticipation in the design of the desired systemic uncertainty of IoT networks should be considered as an instrument to direct the future by imagining it as a way to achieve the desired state. The model of the anticipatory management of systemic uncertainty in IoT networks, proposed in the publication, helps to anticipate problems before they become reality by minimizing the delusional sense of the power of determinism, according to which the imagination of the future should be based only on past, “certain” data.

Desired uncertainty should influence the degree of desired decisions made by the IoT system. The desired uncertainty is either a combination of available uncertainties based on available knowledge levels and future types (domains 1–4 from the UKAF matrix) or it is derived from a single type of future and knowledge (e.g., the fourth domain from the UKAF model). In the analyzed model, an IoT system cannot just be an impersonal autonomous technological sensor entity. It must be a system coupled with a human element (consciousness). It is the social construct that should define for the system whether and what anticipatory actions (passive, reactive, proactive, interactive, or a combination thereof) are desirable under given conditions.

One of the byproducts of public engagement with foresight research methods is a greater sense of self-confidence about an uncertain future. Confidence should be reflected by anticipating the challenges involved, in the form of end results of the used methods [79].

Predestined for anticipatory action is not only the social aspect but also the technological sphere on which the infrastructure of IoT systems is based. The proposed model can be an element of anticipatory artifact of IoT systems with built-in mechanisms of anticipatory uncertainty. There is a kind of analogy to preventive actions based on augmented intelligence solutions [88], or on quantum physics in which the effect can precede the cause [89], or on classes of controllers with forward coupling [8].

Uncertainty (along with ambiguity) is an inherent and even useful aspect of the complex future. Instead of ignoring uncertainty, we should detect it and moderate it to a desired state by, among other things, considering the future and knowledge in their alternative forms.

There is a risk of failure of the systems in question (in this case IoT) caused by a neglect of uncertainty. Potential failures can result from ignorance of model structures and parameter values, errors in the data used to control the model, and errors in the data used to evaluate the model. Each of these potential sources of failure may involve unknown unknowns, as well as measurable uncertainties [6]. In addition, one must be aware of potential side effects as a structural feature of anticipatory systems [11].

One approach that can help view uncertainty as a resource rather than a significant obstacle to planning is to implement a concept based on creating a desirable future through the acquisition of the social construct of futures literacy, i.e., the capacity to know how to imagine the future, and why it is necessary [90]. This is a very promising area for further potential research. In this publication, it has been partially exploited by including in the analysis of uncertainty a typology of futures, but this is only one of the very many elements of this concept.

It should be emphasized that the anticipation that is considered in this article refers to the so-called weak type of anticipation and to overt anticipation. The weak type of anticipation is based on the model [11]. Overt anticipation is characterized by the awareness of one’s system [8]. In the case of this paper, this refers to the awareness of a social construct.

Except for simple systems, no model can represent all the relevant system properties and/or interactions with other systems [11]. In order for the proposed model to have a chance to work effectively, it is necessary to carefully plan and build a socio-technical environment that, on the one hand, has a sense of high awareness about the operation of IoT systems and the associated uncertainty, and on the other hand, operates with an unlimited and open sense of the future.

The ideal approach would be to create a strong anticipation type, understood as a coupling between the system and anticipation types and its environment, with elements of implicit anticipation—inherent in the functioning of a given system [8,11].

Anticipation theory is still in its early stages of development. It lacks a unified conceptual language for theorizing and operationalizing anticipation to facilitate interdisciplinary understanding [11]. This article seeks to partially fill this gap.

The author’s future research based on the conceptual modeling presented in this article and in his other publications e.g., [20,21] will be conducted in real, practical environments—among the stakeholders of IoT projects (researchers, decision makers, and practitioners). Research will focus on the following research problem: on what knowledge and what approach to the future/anticipation do IoT stakeholders deal with uncertainty? The aim of such research will be to contribute to the methodological and epistemological discussions on how to manage systemic uncertainty in the decision-making anticipatory context of IoT in the field of Industry 4.0.

The challenge for further research is to treat time in a qualitative way. This will result in uncertainty, future, and knowledge having different characteristics than a quantitative (linear) approach based on other paradigms. Treating time in a linear way relies on a misconception about a particular future as that point in time that is certain (but only seemingly).

The complexity and associated uncertainty are also influenced by the level of evolution of the Internet itself, in which the IoT network is its fourth generation. A new construct is emerging on the horizon in the form of the “web of thoughts” [91,92], which introduces a new, very interesting theme of anticipatory uncertainty analysis, especially since an anticipatory system is a system capable of predicting its own evolution [78].

## Figures and Tables

**Table 1:** The main ideas discussed in this paper.

Authors	Investigated/Signaled Problem	Possible Future Frameworks to Solve the Problems Presented in the Literature	Author’s Proposal to Solve the Problems
P. Cofta, K. Karatzas & C. Orłowski [4]	The most significant challenge, when managing IoT networks in which uncertainty is an integral component, is to change uncertainty in accordance with the changes in social needs on which the functioning of IoT networks is based.	A key challenge in this area is therefore the organization of the process of such changes.	The organization of the process of changes in social needs can take place through anticipatory systems, based on selected foresight research methods.
R. Poli [8]	It is difficult to predict the future, which is a result of, among other things, ever-increasing complexity and uncertainty. Complex systems are inherently uncertain.	Anticipation is a response to complexity. A proper understanding of anticipation requires the adoption of an innovative conceptual framework.	An innovative conceptual framework for anticipating complex systems should focus on a complex approach, which may be based on areas built on common spaces characteristic of uncertainties, knowledge, futures, and on an open and flexible methodology of qualitative and quantitative foresight research.
M.A. Öner, S.G., Beşer, & P. Şenoğlu [3]	In anticipatory studies, one of the most salient features of many areas of the social and economic disciplines is uncertainty.	There is a risk of failure of the systems in question due to neglect of uncertainty [6].	Instead of ignoring or eliminating uncertainty, we should detect it and moderate it to a desired state by, among other things, considering the future and knowledge in their alternative forms.
K. Selkirk, C. Selin, & U. Felt [16]	There is a wide range of knowledge, both explicit and implicit in different time frames, influencing the formation of different types of futures and perceptions about them.	The analysis of different futures requires new experiences, new ways of combining known elements and different tools supporting this type of reflection.	The analysis of different futures and varieties of knowledge can be greatly enriched by including in this research the analysis of uncertainty at its various stages.

## Data Availability

Data available on request because of restrictions.
